# Supplementary figures and images for: High prevalence of lipopolysaccharide mutants and R2-pyocin susceptible variants in Pseudomonas aeruginosa populations sourced from cystic fibrosis lung infections
Source: Microbiol Spectr. 2023 Oct 25;11(6):e01773-23. doi: 10.1128/spectrum.01773-23 (PMC10714928; doi:10.1128/spectrum.01773-23)

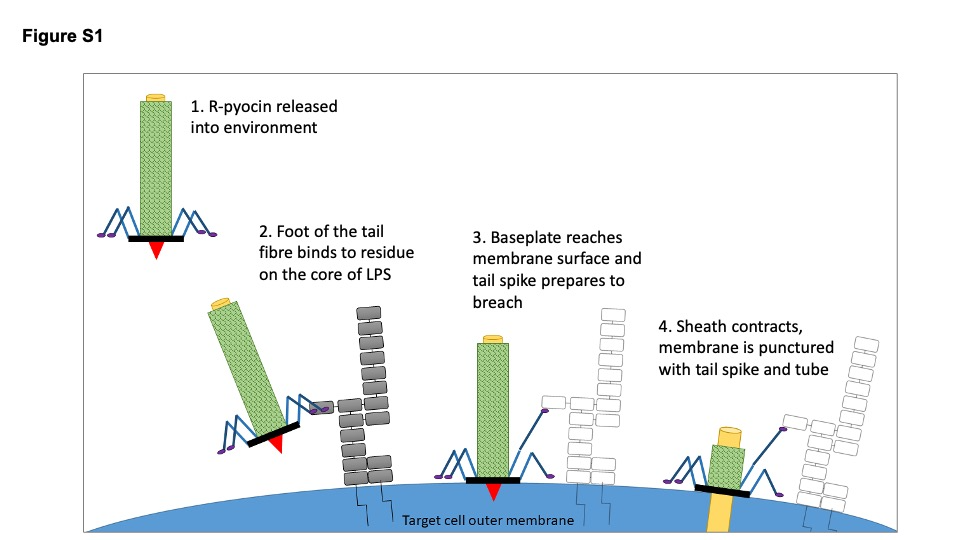

Supplement: Fig. S1 — R-pyocin binding to LPS receptor and puncturing target cell membrane. [file spectrum.01773-23-s0002.tif]

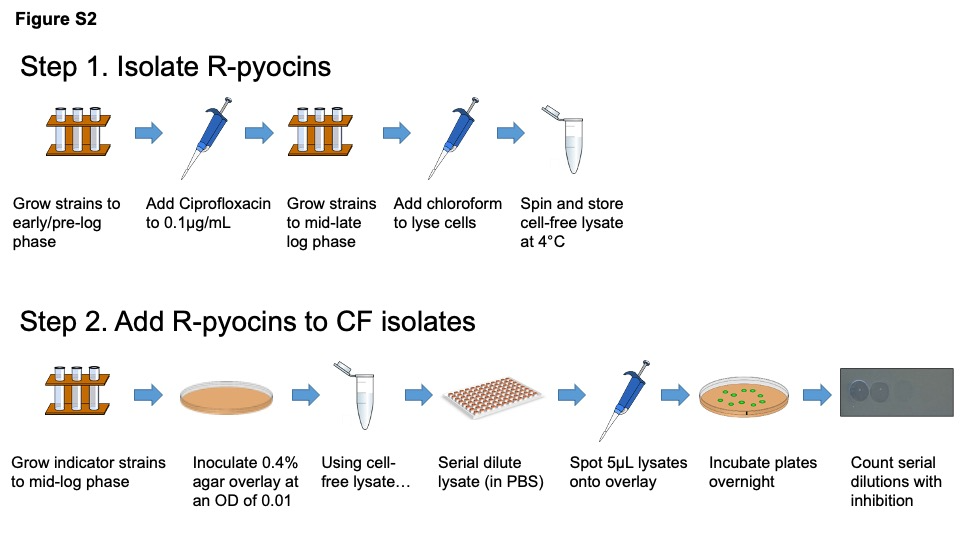

Supplement: Fig. S2 — R2-pyocin lysate collection and susceptibility testing procedure. [file spectrum.01773-23-s0003.tif]
