# Supplementary material for: High prevalence of lipopolysaccharide mutants and R2-pyocin susceptible variants in Pseudomonas aeruginosa populations sourced from cystic fibrosis lung infections
Source: Microbiol Spectr. 2023 Oct 25;11(6):e01773-23. doi: 10.1128/spectrum.01773-23 (PMC10714928; doi:10.1128/spectrum.01773-23)
Supplement: Supplemental legends — Legends for Fig. S1 and S2 and Data Set S1. [file spectrum.01773-23-s0004.docx]

**Supplemental legends**

**Figure S1. R-pyocin binding to LPS receptor and puncturing target cell membrane.** When released into the environment by producing cells, R-pyocins bind to target cells by recognizing core residues of the lipopolysaccharide (LPS) on the outer cell membrane. After tail fiber binding, the baseplate dissociates, initiating sheath contraction and driving the iron-tipped tube through the cell surface, killing the target bacterium.

**Figure S2. R2-pyocin lysate collection and susceptibility testing procedure.** R-pyocins are isolated from *P. aeruginosa* by growing strains to early logarithmic growth, adding sub-minimal inhibitory concentration (MIC) ciprofloxacin, and lysing cells with chloroform after several hours for maximum R-pyocin production. Cultures are then centrifuged, separating into phases, allowing for the collection of cell-free R-pyocin-containing supernatant (lysates) to be stored. R-pyocin lysates are serially diluted and spotted onto soft agar overlays to test the susceptibility of strains of interest.

**Supplemental dataset 1.** Detailed description of bacterial strains, average susceptibility and LPS scoring used in this study.
